# Supplementary material for: Are carbon emissions trading and green financial instruments synergistic? -Comprehensive quantitative research based on content analysis
Source: PLoS One. 2024 Mar 7;19(3):e0298601. doi: 10.1371/journal.pone.0298601 (PMC10919601; doi:10.1371/journal.pone.0298601)
Supplement: S1 Data — (ZIP) [file pone.0298601.s001.zip › Data/地级市Z_F工作报告.zip!/╣π╢1⁄2/╢1⁄2▌╕╩╨/└┤╫╘╣1⁄2╓┌║┼í╛┬φ┐╦╩2╛▌═°í┐_2016.docx]

各位代表：

现在，我代表市人民政府，向大会报告政府工作，请予审议，并请市政协各位委员和其他列席人员提出意见。

2015年工作回顾及“十二五”发展成就

过去一年，在省委省政府和市委的正确领导下，市政府以党的十八届三中、四中、五中全会和习近平总书记系列重要讲话精神为指导，认真贯彻省委十一届四次全会和市委十三届五次全会精神，紧紧围绕“三个走在前列”目标，主动适应新常态的发展要求，扎实做好各项工作，较好地完成了市第十五届人大五次会议确定的年度目标任务。

在经济形势异常严峻的一年里，我们克难奋进，推动全市发展实现了稳中有进。初步核算全市生产总值6275亿元，同比增长8%，与全省持平，快于全国平均水平。市一般公共预算收入518亿元，增长10.2%。进出口增速在全国外贸总额前五名城市中排名第一。农村集体资产增资减债创下历史最优水平。东莞成为全国非省会地级市中第四个各项存款余额破万亿的城市。

在转型升级持续攻坚的一年里，我们聚焦创新，切实增强了科技动力和改革活力。创新驱动发展战略深入实施，全市高新技术企业增加近三成，科技企业孵化器和新型研发机构建设成效明显，“机器换人”如火如荼，工业技改投资大幅增长，首辆新能源汽车下线，东莞前沿技术研究院参与研制的光启“云端”号智慧城市空间信息平台系统完成全球商用首飞。改革创新和工作创新亮点纷呈，商事制度改革持续领先，国家新型城镇化综合试点、“三互”大通关和项目直接落地等改革有效突破。东莞成为珠三角国家自主创新示范区和国家可持续发展实验区。

在全面落实国家战略的一年里，我们争先创优，进一步提升了东莞城市形象和国内外影响力。率先对接国家战略，制定东莞制造2025战略，为东莞制造赢得了新的美誉度。主动布局新丝路拓展贸易版图，对“一带一路”沿线国家出口大幅增长，产业影响力不断提升。加博会、海博会、智博会、漫博会、苏迪曼杯、国际科技合作周等一系列重大展会和赛事活动的成功举办，充分展现了东莞的经济活力和城市魅力。东莞第三次蝉联全国文明城市，荣膺国家森林城市，获评民生周刊全国十大“洗肺”城市，在全国地级市民生发展百强市中排名第七。在北师大发布的地方政府效率研究报告中，东莞再次排在国内104个重点城市第一。

过去一年，我们主要抓了以下工作：

——制定东莞制造2025战略，大力推动“机器换人”和智能装备制造业发展。积极对接中国制造2025，全面推进智能制造、服务型制造、创新制造、优质制造、集群制造、绿色制造“六大工程”。先进制造业、高技术制造业、民营工业增加值分别增长8.5%、10.2%和16.7%。大力实施“机器换人”，申报项目831个，总投资66.9亿元，其中莞产设备占比34.7%。在“机器换人”的带动下，全市工业技改投资增长85.6%，工业投资增长26.6%，占全社会投资比重提升至34.8%。大连机床、省智能机器人研究院等落户东莞，松山湖国际机器人研究院加快建设，全市拥有智能装备制造企业400多家，机器人产业集群初具规模。

——大力实施高新技术企业“育苗造林”行动和科技企业孵化器“筑巢育凤”计划。出台创新驱动发展“1+N”扶持政策。创办创新驱动讲习所。用好高企和研发费用加计扣除等税收优惠政策，为企业减税28.4亿元。全市高企增至986家，获省高企扶持资金4.2亿元，均排全省地级市第一。新增新型研发机构3个、省级创新科研团队4个。发明专利申请量、授权量分别增长61.6%和72.1%。企业国家重点实验室实现零的突破。鼓励企业利用旧厂房等建设孵化器，新增各类企业孵化器13家，总数达36家，在孵企业和项目1000多个，累计毕业企业300多家。预计研发经费支出增长14.8%，占GDP比重2.3%，增幅居全省前列。东莞初具高科技城市雏形。

——推动“四新”经济蓬勃发展。加快生产柔性化、信息化改造，75.6%的规上工业企业具备按订单自动排产和动态调度能力，涌现出一批互联网+家具定制（服装定制、数字印刷）等生产制造典型。加快打造全国智能手机创新研发基地，全市手机年出货量2.6亿台，增长15%。生产性服务业加快发展，电子商务交易额3390亿元，增长16.9%。快递业务收入位居全国地级市第二，获批国家物流标准化试点城市。社会消费品零售总额2154.7亿元，增长10.9%。大力支持企业上市，新增境内外上市企业3家，总数达32家；新增全国股转系统挂牌企业45家、区域性股权交易市场挂牌企业100家，均居全省地级市第一，资本市场的“东莞板块”初现。

——着力打造“一带一路”重要节点城市。组团赴俄罗斯、南非、埃塞俄比亚等国家开展经贸交流活动，拓宽与“一带一路”沿线国家的合作。成功举办海博会，专业采购商人次、签约金额分别增长69%和15.5%。石龙中俄贸易产业园在两国元首见证下正式签约。粤满俄中欧班列启动试运行。粤新欧国际铁路联运专列提升到每周2-3班。石龙铁路货物吞吐量增长102%。东莞全港集装箱吞吐量335万标箱，增长15.7%，其中虎门港250万标箱，比2011年的16万标箱增长近15倍，从全省第9位跃至第3位。东莞全港总货物吞吐量1.3亿吨，在全省排名第4。与德国乌波塔尔市结为友好城市，与韩国金浦市、埃塞俄比亚亚的斯亚贝巴市等签署友好城市备忘录。中马友谊园开园。全市外贸出口增长6.9%，其中对“一带一路”国家出口增长25.2%。

——以“三互”大通关和项目直接落地等为抓手争创更多制度红利。在全国率先启动“三互”大通关，通关时间、费用节省一半以上。项目直接落地试点增至40个，动工项目审批普遍缩短3-6个月。住所登记管理、企业集群注册改革全面铺开，多证联办拓展到所有企业。“三证合一”、“一证一码”改革正式启动，在全国地级市中率先实施全程电子化工商登记，全市市场主体突破70万户，稳居全省地级市第一。设立首期20亿元、总规模200亿元的政府基础设施和公共服务投资基金。组建市交通投资集团、科技金融集团。创建全国农村综合改革示范试点。扎实推进医疗、公交和不动产登记等改革。国家新型城镇化综合试点工作开局良好，相关经验得到上级肯定和推广。

——加快重大项目、重大平台和重要基础设施建设。每月召开重大项目督促协调会，推动重大项目建设实现投资397.5亿元，增长15.7%，带动全市固定资产投资1446.5亿元。实施内外资统筹招商，建立“项目源”大数据，设立驻深圳、北京产业合作联络处和驻美国经贸代表处，新引进57个总投资983.6亿元的重大项目。编制完成城市总体规划（2016-2030年）。松山湖生态园统筹发展机制进一步理顺，松山湖高新区在全国国家高新区排名由第50位升至第30位。水乡新城规划完成编制，华阳湖湿地公园建设受上级肯定和社会认可。银瓶合作创新区总体规划获省批准。引进招商局集团合作开发长安新区。省市重要基础设施建设进展顺利，地铁2号线、莞惠城际轨道试验段试运行，散裂中子源一期、江库联网水源配置一期、虎门高铁站周边交通整治、市民艺术中心等一批工程完工。新建投产110千伏及以上输变电工程19项。成功入选“宽带中国”示范城市。整合连片土地3万亩，盘活存量用地2万亩，完成“三旧”改造5300亩。启动美丽港湾建设。建成美丽幸福村居50个、社区公园42个。

——扎实推进国家节能减排财政政策综合示范市工作。投入17.1亿元推动水乡101家“两高一低”企业加快退出。电机和注塑机节能改造量均居全省第一。五大高能耗行业能耗全部下降，单位生产总值能耗下降7.9%。拒批污染项目740个，基本建成7个环保专业基地。启动400公里截污次支管网建设，推进11家污水处理厂新改扩建。启动麻涌环保热电厂建设，完成横沥环保热电厂一期改造。出台生活垃圾处理生态补偿机制，城镇生活垃圾无害化处理率达100%，农村生活垃圾收运处理全省考核排名第一。启动臭氧污染防治三年行动计划，完成黄标车淘汰任务，补贴推广应用新能源汽车。空气质量达标天数上升14.4个百分点。

——切实加大公共安全管理力度。深入开展“3+2+2”专项行动，严厉打击各类突出违法犯罪，破获一批大案要案。全市刑事案件下降8.7%，破案率上升3.3%。加强矛盾纠纷化解，推进信访法治化建设，劳动仲裁结案率93.8%、调解率90.1%。开展安全生产大检查，深化打非治违和专项整治，对危化品生产经营单位等进行地毯式检查，整改各类隐患1.8万处，全年未发生重特大安全事故。开展环境保护大检查，检查企业3.6万家、处罚环境违法行为3824宗。查处食品药品违法案件1312宗。实现家禽集中屠宰、冷链配送、生鲜上市。数字城管系统试运行。打造城市管理亮点工程102个。拆除违建50万平方米。有效防范H7N9、登革热等疫情。

——全面发展文教体卫等社会事业。启动国家历史文化名城创建工作。开展社会主义核心价值观进企业、进军营活动300多场。入选成为全国5个数字文化馆地级市试点之一。《啊！鼓岭》等一批文艺精品推出并获得好评。高考四项指标连续四年排全省第一。东莞被授予省推进教育现代化先进市荣誉称号，顺利通过全国义务教育发展基本均衡市国家督导验收。省市共同支持东莞理工学院建设高水平理工科大学。成功举办苏迪曼杯赛事，成为2019年男篮世界杯承办城市之一，东莞代表团在省运会上总分和金牌总数名列第三，在省残运会上总分、金牌数和奖牌数均列第一。入选公立医院改革国家联系试点城市，财政补贴医院2840万元，取消公立医院药品加成，减轻群众负担7600万元。推行家庭医生式签约服务，实现医疗责任险公立医院全覆盖，试行微信预约挂号支付。打造“志愿之城”，全市志愿者占常住人口10%。培训应急救护5万人次。提高城乡居民养老金标准和低保标准，向困难群体发放各类补助金1.6亿元。居家养老服务实现城镇社区全覆盖。成立白玉兰创业就业服务中心。发放就业补贴3.4亿元，小额创业贷款规模全省第一。十件实事圆满完成。

——加强区域协作与对口帮扶工作。出台扶持欠发达镇发展意见，在重大项目、基础设施等方面给予倾斜。在全省率先全面推行村级集体经济组织预算制度。农村集体资产网上交易平台面向全国竞价。村组两级纯收入增长9%，资产负债率降至17.2%。超额完成欠发达村组、有劳动能力低保家庭帮扶任务。全部实现对口帮扶韶关、揭阳三年扶贫开发“双到”目标。新疆草湖产业园首期30万锭棉纺项目即将投产，西藏鲁朗国际旅游小镇完成工程进度80%。积极参与四川甘孜州、广西河池、重庆巫山帮扶工作。深化“深莞惠+汕尾、河源”合作。国防动员、统计审计、人口计生、工青妇幼、民族宗教、档案方志、科普法普、气象、打私等工作有效推进。

——以“三严三实”要求推进政府建设。在政府系统开展“三严三实”专题教育，深入查摆整改“不严不实”问题，作风进一步转变。出台加强法治政府建设意见，制定重大行政决策事项听证目录，完成《东莞市城市综合管理条例》（草案）起草，出台首部政府规章《东莞市城市轨道交通运营管理办法》。在中国政法大学发布的法治政府评估报告中，东莞总分位居全国第七、地级市第一。加快网上办事大厅建设，完善进驻事项目录，开通网厅村居办事点，行政审批事项网上全流程办理率98.1%、办结率99.5%。整合部门镇街服务热线，实现12345政府热线“一号通”，服务水平在全国首次城市公共服务热线调查中排名第五、省内第二。

2015年也是实施“十二五”规划的最后一年。“十二五”时期，我们积极应对后金融危机时代的复杂形势，以高水平崛起为总目标，以创新型经济为主攻方向，以加快“三重”建设、打造“三大增长极”、推动科技金融产业融合、深化重点领域改革、营造法治化国际化营商环境等为主抓手，较好地完成了各项目标任务，推动经济社会在科学发展的道路上迈出了坚实的步伐，取得了“一个跨越、两个突破、三个转变、四个提升”的新成就。

“一个跨越”：即城市综合实力实现新跨越。全市生产总值相继突破5000亿元、6000亿元大关，来源于东莞的财政收入突破1000亿元，社会消费零售总额突破2000亿元，进出口总额迈上1万亿元人民币新台阶，金融机构本外币各项存款进入1万亿元俱乐部。全市引进培育一大批关系城市未来发展的重大项目，500亿元企业实现零的突破，100亿元、50亿元企业分别增至12和34个。先后获得全国文明城市、全国双拥模范城、国家园林城市、国家环保模范城市、国家森林城市和国家公共文化服务体系示范区等荣誉称号。

“两个突破”：即区域统筹发展取得有效突破。水乡经济区、大学创新城、银瓶合作创新区“三大增长极”建设全面推进，市镇中心区功能不断完善，以经济区理念加强区域整合、促进协调发展取得了阶段性成效，“一中心四组团”的城镇群格局基本奠定。重点领域改革取得有效突破。330多项莞版改革陆续铺开，东莞以主动改革、自我革命的勇气，在多个重点领域率先突破，十大改革品牌领跑全省全国，激发出更加蓬勃的内生动力和改革红利。

“三个转变”：即发展动力从要素驱动向创新驱动逐步转变。全市创新要素加快集聚，新型研发机构、省创新科研团队、全社会研发投入加快增长，国家高新技术企业增长3倍，发明专利授权量跃居全省第三，规模以上先进制造业、高技术制造业增加值占比分别提高7.4和10.9个百分点，创新对经济增长的贡献率越来越高。开放格局从外向依赖向内外并重逐步转变。全市外向依存度下降25.7个百分点，规模以上工业企业内销比重超过外销，外资和民营“比翼齐飞”、出口和内销“两分天下”、引进来和走出去“双轮并驱”的格局初步形成。经济形态从传统制造向创新型经济逐步转变。加工贸易转型升级成效明显，智能手机、机器人等新产业形成规模，电子商务、现代物流等新业态、新模式生机蓬勃，以创新产业为标志、以新技术和新产品为着力点的创新型经济加快发展。

“四个提升”：即城市品质提升。城市基础设施和配套进一步完善，建成东莞地铁2号线、莞惠城轨试验段、东莞新火车站、虎门高铁站，新建改造高速路、国省道、镇村联网路超过400公里，以篮球中心、“四院一中心”等为代表的综合配套不断丰富，4G网络、WIFI和供水供电、邮政快递服务设施不断完善。生态环境提升。全市新建森林公园、湿地公园16个，建成绿道955.9公里、休闲绿地1287处，内河涌与跨界河污染整治成效明显，空气质量在全国重点监测城市中排名上游。社会治理提升。“黄赌毒”现象得到铁腕整治，“两抢一盗”等案件稳步下降，劳资纠纷、医疗纠纷、群众信访等得到妥善处置，全市未发生重特大安全事故，社会文明程度持续提高。民生水平提升。市财政共投入1160.3亿元用于民生建设，十件实事成为每年民生工程突出亮点，城乡居民收入、基本养老金、城乡救助及福利水平逐年提高，城乡一体、全民享有的社保体系日益完善，教育、医疗、文化、就业、住房等幸福保障网不断加固，社会事业发展水平处于全省全国前列，全市人民在共建共享中得到了更多实惠。

在全市人民的共同奋斗下，东莞“十二五”时期发生了一系列明显变化。我们的天更蓝了，大气污染物浓度逐年下降，空气质量优良天数逐年增加，蓝天白云成为常态。我们的水更清了，东江、运河和石马河口水质不断好转，水乡重现花红水绿的生态环境，不少河涌变身成为市民休闲娱乐的好去处。我们的城更靓了，公共基础设施日趋完善，美化绿化水平大幅提升，走在东莞的大街小巷，处处可见绿、处处是美景。我们的路更通了，高快速路网纵横交错、四通八达，轨道交通建设加快推进，东莞即将迈入地铁时代。我们的城市更安全了，治安案件进一步下降，安全事故进一步减少，群众安全感进一步增强。我们的城市吸引力更强了，越来越多优质企业来莞投资，越来越多高素质人才扎根东莞，每年来莞观光旅游的游客超过2700万人次。我们的经济质量更好了，全市发展调速不减势、量增质更优，东莞正朝着高水平崛起的道路上阔步迈进。

各位代表，“十二五”时期取得的成绩，离不开历届市委市政府打下的坚实基础，是全市上下共同努力的结果，为今后的发展提供了跃升的平台。在此，我代表市人民政府，向全市广大干部群众，向人大代表和政协委员，向各民主党派、各人民团体、社会各界人士，向各驻莞单位、驻莞部队、武警官兵，向所有支持东莞建设与发展的港澳台同胞、海外侨胞和国际友人，表示衷心的感谢和崇高的敬意！

在肯定成绩的同时，我们也清醒地认识到，全市在协调发展、生态环境、社会建设等方面还存在明显短板，发展中面临的深层次矛盾和问题仍然突出：一是经济平稳较快增长的基础还不稳固。后金融危机时代的外部市场环境持续复杂严峻，引进的重大项目全部建成投产、强力支撑经济发展仍需时日，新的增长点有待培育，一些年度部分指标未能完成计划目标。二是发展模式尚未根本转变。经济发展仍是较多依靠传统路径，“四新”经济还未起到决定性的作用，完成经济结构战略性调整还有较长的路要走。三是镇村基层发展面临诸多困难。一些镇街由于招商乏力、用地趋紧等原因导致发展缓慢，部分镇街债务结构有待优化。农村集体经济保持稳定增长压力加大。四是生态建设任重道远。跨界河流污染整治任务繁重，企业环境违法行为仍然存在，引导企业发展循环经济、促进市民低碳绿色消费还需加大力度。五是公共服务供给仍有较大的提升空间。经济发展同步提升了人民群众对公共服务的需求和标准，必须在财力可支撑、可持续的同时，想方设法扩大公共服务供给，逐步提升均等化水平，进一步提高广大群众的幸福感。六是政府系统的作风建设和履职水平仍需进一步提升。一些干部干事创业的激情有所退化，存在多干事不如少犯错的思想，部分领导干部驾驭复杂局面的能力还有不足，企业和群众对行政审批效率、窗口服务质量仍有意见，法治建设、廉政建设仍需进一步加强。我们将积极面对并认真解决这些问题，努力把各项工作做得更好。

“十三五”主要目标及2016年工作安排

“十三五”时期是我国全面建成小康社会的决胜阶段，东莞发展既处于可以大有可为的战略机遇期，也面临着诸多矛盾叠加、风险隐患增多的严峻挑战。我们要看到，外部环境正在发生深刻变化，既有全球经济曲折复苏、新一轮科技革命和产业变革蓄势待发等积极因素，又有贸易保护抬头、地缘政治复杂、制造业部分转移等不利影响，但我们更要看到，国内经济长期向好的基本面没有改变，创新、协调、绿色、开放、共享五大发展理念全面贯彻，中国制造2025、“一带一路”、“互联网+”等战略深入实施，新型城镇化加快推进，全面两孩等政策红利逐步释放，将提供更加广阔的市场空间，孕育形成新的增长动力。特别是中央着眼战略全局，在适度扩大总需求的同时，着力加强供给侧结构性改革，重点推进去产能、去库存、去杠杆、降成本、补短板五大任务，将有效化解产能过剩、债务风险、实体经济成本上升等问题，对于提升社会供给水平、提高全要素生产率有着重大的促进作用，必将为东莞这一国际制造业基地注入新的蓬勃生机。我们要紧紧把握新一轮发展的历史机遇，珍惜近年来狠抓重大项目建设等打下的扎实基础，巩固产业转型升级的阶段成效，强化创新驱动发展的良好态势，再接再厉，奋发有为，努力开创全市发展的崭新局面。

去年12月召开的市委十三届六次全会，审议通过了《中共东莞市委关于制定国民经济和社会发展第十三个五年规划的建议》，提出“十三五”时期我市要以创新驱动发展、对外开放合作、重点改革突破“三个走在前列”为战略重点，以提高发展质量和效益为中心，加快形成引领经济发展新常态的机制体制和发展方式，推动经济结构战略性调整和转型升级，统筹推进经济、政治、文化、社会、生态文明和党的建设，努力建设国际制造名城、现代生态都市，确保率先全面建成小康社会，为全省实现“三个定位、两个率先”的总目标作出更大贡献，迈上率先基本实现社会主义现代化的新征程。《建议》为全市未来五年发展明确了发展目标和工作方向。市政府据此编制了《东莞市国民经济和社会发展第十三个五年规划纲要（草案）》，并提交本次人代会审议。

我们坚信，只要认真贯彻执行上级和市委的决策部署，将“十三五”规划各项工作任务落到实处，东莞未来五年必将呈现出一幅更加美好的发展图景。“十三五”时期我市经济社会发展的基本目标是：经济保持中高速增长，地区生产总值年均增长8%左右。到2018年全市小康指数达到97%以上，率先全面建成小康社会。到2020年地区生产总值超过9200亿元，人均地区生产总值约11万元，建设国际制造名城、现代生态都市取得显著成效，以创新为主要引领和支撑的经济体系和发展模式基本形成，天蓝、地绿、水净的环境成为常态，对外开放的质量和发展的内外协调性明显提高，人民生活水平迈上新台阶，率先基本实现社会主义现代化的基础将更加坚实。

2016年是实施“十三五”规划的第一年。做好今年政府工作，必须以党的十八届三中四中五中全会、中央经济工作会议、习近平总书记系列重要讲话以及省委十一届六次全会精神为指导，坚持创新、协调、绿色、开放、共享五大发展理念，紧紧把握“十个更加注重”的工作要求，认真贯彻落实市委十三届六次、七次全会各项部署，努力实现“三个走在前列”目标任务，推动全市发展稳中有进、稳中向好。综合考虑各方面因素，今年全市发展的主要预期目标是：生产总值增长8-8.5%，人均生产总值增长8%左右，市一般公共预算收入增长9%，固定资产投资总额增长10%左右，社会消费品零售总额增长11%左右，进出口总额增长3%左右；先进制造业、高技术制造业增加值占规模以上工业增加值比重分别达48%和37.3%；研发经费支出占生产总值比重2.4%；国家高新技术企业数量达到1100家；发明专利授权量增长10%；城市居民人均可支配收入、农村居民人均可支配收入分别增长8%和8.5%；居民消费价格指数控制在3%以内。围绕以上目标要求，重点做好几方面工作：

一、在兼顾需求侧的基础上突出抓好供给侧结构性改革，为“十三五”开好局增添新动力

供给侧改革的核心是创造有效需求。东莞作为一个有强大制造能力的城市，应当在供给侧结构性改革当中发挥重要的作用。要在增强三驾马车动力的同时，集中力量推进东莞本土的供给侧结构性改革，通过提高产品质量、创造市场需求，让全国商家都来莞采购，让东莞的产品行销全世界。

着力打响莞货质量品牌。巩固全国质量强市示范城市创建成果，加快提高传统优势产业质量水平，打响虎门服装、大朗毛织、长安五金模具、大岭山家具、茶山食品等区域品牌，提升产品附加值，扩大优质消费供给。大力实施标准化战略，增强东莞企业话语权和东莞产品竞争力。实施技改事后奖补政策，鼓励企业开展增资扩产、设备更新和智能化改造，力争全年技改投资增长20%以上。整合科技、人才等专项资金，优化拨付流程，提升资金使用乘数效应，助力企业提高产品质量和层次。

创新机制有效引导和培育市场。聚焦世界产业前沿和重点领域，前瞻布局可穿戴设备、可见光通信技术、航空航天等新兴产业。扶持有实力的莞企和研发机构开发新技术、新产品，创造新的市场需求。加强新产品的应用展示和市场推广，加快引进生产链上下游企业，逐步形成集群效应。推动军工技术向民用技术转化，引导优势民营企业进入军品科研生产和维修领域，创建军民融合创新发展示范区。

扩大优质服务的有效供给。加快发展文化创意、健康养老、现代医疗等产业。鼓励设立跨境电商体验店。设立旅游产业发展专项资金。深入挖掘工业游、莞香游、古村游、休闲游、生态游等旅游资源，创建国家级旅游景区，抓好茶山南社等4A级景区建设。推动重大旅游项目的洽谈、建设。积极发展都市农业。

推动企业优化结构、做大做强。大力推进市场取向改革，淘汰落后产能，出台政策鼓励优势企业实施强强联合、对外并购和重组整合。大力培育大型骨干企业，认定大型骨干企业30家以上。建立成长型中小企业储备库。新增个转企500家、小升规550家以上。大力推动重大产业项目上马，落实责任、逐月督导、强力推进，确保桥头新技电子等32个新项目开工建设，石碣盈聚电子等17个项目竣工投产。大力招引优质项目，完善招商激励机制，加强与周边城市产业合作，主动接受辐射。加强用地保障，争取整合1000亩以上连片土地10块。

开展降低实体经济企业成本行动。巩固历年减负成果，落实国家和省减负政策，加强扶企措施宣讲，确保政策落到实处、发挥作用。用好地方金融稳定专项资金。积极推进已建房屋补办产权，妥善解决历史遗留问题，盘活企业沉淀资产。鼓励优质企业发债融资。推动贷款更多向制造业倾斜。做好经济运行监测与统计。

二、把握珠三角国家自主创新示范区建设机遇，着力创建国家创新型城市

发挥自主创新示范区、自贸试验区政策溢出效应。围绕东莞创新中轴线，统筹松山湖（生态园）核心园区，散裂中子源、大学创新城以及寮步、大岭山、大朗、横沥、东坑、企石、石排、茶山、石龙等9个镇，规划建设东莞自主创新示范区。加快完善自创区配套政策、规划体系，积极向上争取在东莞自创区优先推广复制自贸区政策，承接自贸区溢出效应和制度红利，形成政策叠加效应。办好全省专业镇建设现场会，推广横沥协同创新模式，打造组团式区域创新体系。

进一步强化企业创新主体地位。继续推进“育苗造林”行动，力争年内高企总数达1100家以上、高企培育入库企业700家以上。每年安排一定比例的新增用地指标，优先满足高企及高企培育入库企业用地需求。鼓励企业开展核心技术攻关，实施重大科技专项，争取国家和省级科技项目，新设市级以上重点实验室、技术中心等研发机构30个。试点科技创新券，推动企业设立研发准备金，引导企业加大研发投入，力争全年R&D投入增长10%。

加快推进新型研发机构和高水平理工科大学建设。赋予新型研发机构对科技成果更大的自主使用权、处置权和收益权。探索科技用房产权分割转让。搭建新型研发机构与行业协会、企业的沟通合作平台，组织技术成果展示对接活动，让新型研发机构更接地气，成为服务转型的助推器。五年投入35亿元、以超常规举措支持东莞理工学院创建高水平理工科大学，面向全球重金招揽人才，加快建设成为以智能制造学科群为龙头的区域创新发展高地，力争2020年进入全国理工类院校百强。引进国内外一流大学来莞设研究生院。探索建立科研院所、高校和企业创新人才互动交流机制。

构建科技创新融资新模式。市财政投入10亿元设立产业投资基金，撬动社会资本超过40亿元，培育引导新兴产业发展。与深交所、上交所等合作，强化企业上市、新三板挂牌、资产证券化等指导培训，推动企业进军资本市场。出台优惠政策，吸引各类风投、创投集聚，定期举办创新成果与创业资本对接会。建设统一平台，整合银企对接、征信查询等功能，为企业信用融资提供支撑。加快组建市属金融控股集团。支持设立地方保险机构。

三、大力实施“机器人智造”计划，打造有全球影响力的先进制造基地

着力建设中国机器人产业先行市。鼓励重点行业开展“机器换人”应用示范，为企业提供全流程解决方案。省政府和市政府共同出资2亿元设立融资租赁专项资金，与国开行开展“零首付、零门槛”技改信贷计划，解决中小企业“机器换人”资金难题。加强机器人创新体系建设，重点突破机器人整机和关键零部件技术。调查摸清食品、服装、家具、电子等行业实际需求，集中突破“机器换人”行业共性问题，努力形成可复制推广的产业化成果，提升莞产设备采购率、使用率。大力发展工业机器人、服务机器人、特种机器人，有针对性地开展点对点招商，引进若干家有国际国内影响力的机器人龙头企业，加快形成覆盖本体、关键零部件、系统集成商、服务提供商的机器人产业集群，力争机器人产业产值增长30%。

大力推动“东莞制造+互联网”融合发展。鼓励企业开展生产流程智能化改造，推广数字化工厂等生产模式，全面提升劳动生产率。推广运用工业云服务平台，对租赁企业给予最高20万元的补助。与阿里巴巴集团合作建设“阿里巴巴—东莞产业带”。大力鼓励电子商务发展，加强专业人才的招引和培育，支持设立国际邮件互换局兼交换站，认定一批电子商务示范企业，建设东莞跨境电商中心园区，推动外贸综合服务企业和物流企业电商化改造，打造具有全国影响力的自营电子商务平台。抓好大数据综合服务平台建设，整合各类信息资源，进一步优化对企业的监测和服务。

大力推动“东莞制造+四新”融合发展。研究制定扶持“四新”经济发展政策。鼓励服装、家具等传统企业通过手机APP等，与消费者精准互动，建立柔性化、定制化生产模式，打响“东莞定制”品牌。办好国际工业设计大赛，加快建设一批国家级、省级工业设计中心。落实扶持会展业发展各项措施，推动展会迈向品牌化、国际化，促进与制造业融合发展。

大力推动“东莞制造+双创”融合发展。出台众创、众包、众扶、众筹平台建设政策。鼓励在“三不变”的前提下，通过“三旧”改造建设科技园、众创空间和示范基地。设立孵化器扶持专项资金。实施莞港澳台科技创新创业联合培优行动计划。办好各类创客嘉年华活动和创新创业大赛。鼓励企业员工内部创业，支持青年学生初始创业。将创业小额贷款最高提至20万元。对创新创业领军人才给予最高200万元的启动资金扶持。

四、建设“一带一路”海陆联动桥头堡，拓展对内对外开放新空间

加快广东铁路国际物流基地和水铁联运大通道建设。推动粤新欧、粤满俄、中韩快线等国际铁路班线跨越式发展，加快建设中俄物流产业园和石龙口岸功能区，打造广东陆上丝绸之路重要起点。加快建设虎门港9、10号泊位，开辟东南亚等国际航线，打造海上丝绸之路的重要航运和物流节点。实施虎门港与石龙铁路货运站海铁联运，构建连接“一带一路”国家的国际物流大通道。大力鼓励企业走出去，支持华坚埃塞俄比亚工业园、巴中贸促会总部项目建设。继续办好海博会、加博会，不断扩大与海上丝绸之路沿线国家和地区的经贸合作。

加快对外贸易优化升级。实施优进优出战略，增强一般贸易核心竞争力，深度开发多元化国内外市场，保持外贸进出口稳定增长。引导外资企业更加注重创新发展，争取新培育100家外资高新技术企业、250个外资企业研发机构、3家外资上市企业。申报建设虎门港综合保税区，建设清溪保税物流中心（B型）。推进水运口岸“三互”大通关系统应用。加快国际贸易“单一窗口”建设。

大力扶持内源型经济发展。完善市属企业产权管理、薪酬考核、预算评价等制度。推动市属资产优化配置，采取股权出让、职工持股、引入战略投资者等方式，实现产权主体多元化。开展市属企业公司制改造，支持和引导条件成熟的企业组建或改组为国有资本投资公司。完善先进制造业项目数据库，定期举办对接活动，引导民间资本与先进制造业项目对接。推进“686”上市后备企业培育工程，鼓励优质民营企业股份制改造和上市。

五、以实施城市总体规划（2016-2030年）为契机，促进城市协调绿色发展

以地铁建设重构城市空间格局。推动地铁2号线、莞惠、穗莞深城轨早日开通运营。开工建设地铁1号线，加快佛莞城轨建设，抓紧中虎龙城轨前期论证，开展莞深快轨建设规划。谋划轨道交通沿线开发利用，推进周边TOD开发。科学预测市民出行方式转变和交通流量的变化，加快常规公交、有轨电车与地铁无缝衔接，完善公交线网覆盖，逐步形成以地铁为骨干、常规公交为主体的公共交通体系。试点建设公共自行车租赁系统，优化绿道配套设施，完善城市慢行系统。主动对接国家、省铁路建设规划，争取赣深客专在莞设点。加强与深圳、广州等周边城市轨道交通对接。

以水乡、银瓶合作创新区建设为突破促进协调发展。加快水乡新城及周边配套设施建设，打造水乡文化创意产业样板区，试点推动创意设计与制造业融合发展。加快银瓶合作创新区基础设施建设，推动大连机床等重大项目投产，开工建设5个重大项目，争取新引资50亿元。与招商局共同加快开发长安新区，打造“一带一路”国际合作示范区。提高财政困难的欠发达镇街税收增收分成比例。将市对镇街的财力性转移支付提高到20亿元。加大对经济发展滞后镇街基建投资的分担补助。加大市内对口帮扶力度，力争60%的欠发达村两级经营性纯收入达300万元或增长20%以上，60%的帮扶低保劳动力稳定就业。基本完成农村土地承包经营权确权登记颁证。推动“深莞惠+汕尾、河源”经济圈建设，加快区域一体化进程。

推动基础设施再升级。启用“无线东莞”WiFi，建成2万个无线接入点，实现全市主要公共区域免费WiFi全覆盖，打造全国第一批“无线地级市”。与光启合作建设智慧城市空间信息平台，全面提升环境监测、智慧交通、应急管控等管理能力。推进虎门二桥、梨川大桥、莞番高速东莞段、莞深高速黄江出入口等公路桥梁建设及改造工程。加快220千伏双岗变电站等17项电网、水利工程建设。以黄沙河流域为试点推进海绵城市建设。探索推进地下管线综合管廊建设，打造管线高速。

完成节能减排财政政策综合示范市建设任务。开展千家工业企业节能低碳行动，扶持一批重点用能单位开展节能技术改造。争取成为国家绿色供应链试点工作示范城市。推进新一轮治污行动，建立水污染防治项目库，加快截污次支管网建设，启动现有污水厂提标改造。狠抓茅洲河、石马河等重点流域及内河涌污染整治，治理松木山水库和同沙水库。大力推进臭氧、PM2.5等协同治理。推动大型工业燃煤锅炉和区域性集中供热改造。在石碣、麻涌试点开展土壤污染修复，规划建设建筑余泥渣土消纳场。推进桥头大东洲、清溪罗马等垃圾填埋场整治，抓好麻涌、横沥环保热电厂建设改造，力争两年内实现原生生活垃圾全焚烧进而实现零填埋。新建美丽幸福村居50个，探索推进连片特色示范区试点项目建设。进一步完善森林公园、湿地公园配套。

六、进一步扩大公共产品和公共服务供给，让东莞人民共享改革发展成果

加快构建社会治理立体网格化体系。探索在社会治安、安全生产、食品安全、城市管理等全领域推行网格化管理，划分若干网格，明确治理责任，利用互联网等科技手段强化治理，力求取得叠加治理效应。保持严打高压态势，重拳打击“黄赌毒”、“两抢一盗”等违法犯罪。加强公路检查站、治安卡点和“智能天网”建设应用，强化流动人口及出租屋管理。开展以案说防教育，倡导人文关怀，预防和减少各类违法犯罪案件的发生。落实安全生产责任体系，建设“智慧安监”信息化平台，健全镇街（园区）专职安全员队伍，加强危化品整治和特种设备监管。强化气象预测、预报、预警、预案和值班工作。加大打假工作力度，强化农产品日常检测监督。加强劳资矛盾分析和化解，加大恶意欠薪打击力度。完善信访工作机制，依法解决群众合理诉求。实现村（社区）公共法律服务站、消防站全覆盖。启动数字城管系统二期建设。落实新增违法建设追责机制，力争实现违建“零增长”。

办好人民满意的教育。落实异地高考政策。通过积分制新招收3.3万名随迁子女就读义务教育阶段学校。出台实施向民办学校购买学位政策，市财政补贴7.1亿元支持民办教育。加快慕课教育平台建设，新增微课1万节、推送2万节，选定100所学校开展慕课教学试点。推动30%教师利用慕课学习。积极创建省现代职业教育综合改革示范市。

推进健康东莞建设。出台城市公立医院综合改革配套文件。建立全市统一的网上预约挂号平台。开展免费的孕妇唐氏综合征、新生儿耳聋基因筛查。放开医师多点执业。建立分级诊疗制度。实施全民健康促进行动，巩固篮球、羽毛球等体育基础，发挥绿道、公园众多的优势，组织开展长跑、徒步、自行车等户外赛事，打造珠三角户外运动和体育旅游基地。办好松山湖国际马拉松、欧亚全明星乒乓球对抗赛等重要赛事。

提升城市文明程度。深入推进社会主义核心价值观建设，实施文化惠民工程，加强优秀传统文化普及教育，推动传统媒体和新兴媒体整合发展，建立健全公益广告发布长效机制。开展千场家庭教育大讲堂进社区活动，加强和改进未成年人思想道德建设。创建全国版权示范城市。争创全国文明城市“四连冠”。

全面推动社会事业发展。推进社保、住房公积金扩面征缴。制订基本公共服务“同城同待遇”批次清单。贯彻实施全面两孩政策。落实新生育保险政策，生育医疗费用按实际核付，生育津贴与单位月平均工资挂钩。将基本医疗保险年度最高支付限额提高至30万元。提高低保家庭食品、燃气及水电补助标准。加强人才队伍建设，建立柔性引进海外专家工作机制，启动技能人才五年行动计划。建设新妇女儿童活动中心。抓好残疾人保障服务。继续做好对口帮扶韶关、揭阳，以及对口支援新疆兵团第三师、西藏林芝市巴宜区、四川甘孜州、广西河池、重庆巫山等工作。

七、全面建设法治政府和服务型政府，不断提升政府公信力和执行力

着力建设全省全国领先的法治城市。用好地方立法权，加强对水乡生态建设、饮用水源水质保护等的立法调研和草案起草工作。完善政府规范性文件制定计划和流程。制定行政执法细则、裁量标准和操作流程，确保规范文明执法。完善政府法律顾问制度。严格落实重大行政决策程序规定，推动公众参与决策常态化。打造阳光政务，加大对镇街公开财政预决算信息的指导力度，全面公开部门预决算信息和“三公”经费支出信息。

打造更廉洁、更高效的政务环境。严格执行中央八项规定，加强政府系统廉政建设。深化行政审批制度改革，进一步压减和下放行政审批事项，启动行政审批标准化建设。推动商事制度改革继续走在全国前列，进一步推进前端便利化登记，全面加强后续监管。深化项目直接落地改革，简化审批推广至所有项目。优化网上办事大厅功能，推动更多服务事项实现网上全流程办理。全面落实权责清单制度，形成市镇两级权责清单。创新事业单位管理体制，探索公益三类、经营服务类事业单位的脱事转企改革，开展事业单位法人治理结构试点。

进一步提振干事创业的精神状态。以严格的问责倒逼机关作风持续转变，继续开展第三方评估，扩大暗访和作风评议范围，曝光政府系统的落实不力、庸懒散拖、吃拿卡要等问题，进一步强化警示教育效果。以强有力的督导倒逼工作加快落实，推动政府系统提振奋力攻坚、改革突破的精气神，一件一件工作抓落实，一个一个难题求突破，一步一个脚印谋实效，高质高效地完成全年各项目标任务。

各位代表！新的航程已经开启，新的任务光荣艰巨。让我们紧密团结在以习近平同志为总书记的党中央周围，在中共东莞市委的坚强领导下，紧紧依靠全市人民，真抓实干，开拓进取，锐意创新，奋力夺取“十三五”新胜利，在东莞高水平崛起的道路上继续阔步前进！
